# What led to upsurge of COVID-19 cases in Malawi? Public health perspective

**DOI:** 10.11604/pamj.2021.38.269.28338

**Published:** 2021-03-16

**Authors:** Chancy Skenard Chimatiro, Precious Luwidzyi Hajison

**Affiliations:** 1 Ministry of Health, Department of Administration, Phalombe District Health Office, Phalombe, Malawi,; 2 School of Public Health, Department of Health Systems and Policy, College of Medicine, University of Malawi, Blantyre, Malawi,; 3 PreLuHa Consult, Namiwawa Street, Newroard Location, P.O Box 703, Zomba, Malawi

**Keywords:** COVID-19, upsurge, low risk perception, unchartered roots, testing sites

## Abstract

The health system in Malawi has been overwhelmed with the growing number of COVID-19 cases during second wave of attack. The number of confirmed cases and case fatality rate has significantly increased as compared to the first episode between the months of January to June 2020. Majority of cases reported are through internal transmission, with no history of international travelling. Those in urban areas are most affected as compared to rural areas. Strict preventive measures with multi-sectoral collaboration are urgently required to curb the further spread of the disease. This paper discusses some of the factors that have led to upsurge of COVID-19 cases in Malawi from public health perspective.

## Perspectives

The first case of the pneumonia-like disease of unknown etiology was reported on 31^st^ December 2019 in Wuhan City, Hubei Province of China [1]. As of 3^rd^ January, 2020, a total of 44 patients were reported to World Health Organization (WHO) by authorities in China. It was later indicated that the disease was caused by virus isolated on 7^th^ January, 2020. Globally, its genome was shared on 12^th^ January, 2020. The virus became to be known as COVID-19. Further, it is believed to be spillover of an animal coronavirus which was later adapted through human-to-human transmission [2]. The virus is highly contagious and rapidly spread throughout the world within a short period of time.

The government of Malawi approached the pandemic in a positive way to reduce its effect. Special Cabinet Committee on COVID-19 was appointed responsible for the provision of policy guidance [3]. The country declared COVID-19 as a state of disaster. Vending, public gatherings including religious gatherings, wedding ceremony, pubs were restricted. Further, schools were closed in order to combat the virus. There was no complete lock down in the country as compared to other neighboring countries like Zimbabwe [4]. The failure to ban some social activities and complete lock down can be attributed to the unstable political situation the country experienced that time. There was tension as some quarters of the population felt that lock down was deliberate move by the then ruling party to shun away from the court ordered Fresh Presidential Election (FPE). Some prominent political figures were publicly refuting the presence of the virus in the country. This instilled doubt in majority of people in the country to whether the pandemic was genuine or not. Until when the elections were over, the country united and agreed that indeed there is COVID-19 pandemic which requires attention.

**Early public health measures taken:** Malawi, like any other country affected with the pandemic, followed and adopted public health measures issued by the WHO. According to WHO, public health measures include personal protective measures (hand hygiene, respiratory etiquette), environmental measures, physical distancing measures, and travel-related measures [5]. At the very onset of the pandemic in the country, deliberate efforts were put in place to strengthen surveillance at all levels. Each District Council, a COVID-19 coordinating office was established. Public Health Emergency Committees (PHEMC) and Rapid Response Teams (RRT) were also formed. Several capacity building trainings to these structures were conducted. To enhance coordination at national level, especially on monitoring of travelers, a network of incident managers in all districts were formed. Data for all travelers through formal ports were shared with all incident managers scattered in all councils for follow up and monitoring.

In terms of public health measures, the first approach that was adopted by majority of people in Malawi especially within urban areas was on personal protective and hygiene. Hand washing facilities and sanitizers were placed in various places like markets, banks and shops. During this stage, with high degree of fear, majority of people used the hand washing facilities frequently without being forced or reminded. Many shops had an attendant on the doorstep ready to sanitize every customer who may enter. Further, body temperature for customers was taken regularly to detect those that might have high fever. Majority of people used face masks to cover their mouth and nose to avoid further spread of the virus. All those who were using public transport were required to wear face masks apart from being hand sanitized by the drivers.

On environmental measures, though not intensified, Malawi government emphasized on continuous spray of public offices. Notices were issued about close of some important places for disinfection. This was done in most of the government and public offices. Further, government directed that all workers should be on shifts except frontline health workers. During early days of the pandemic, majority of those who were infected were reported to have an international travelling history. The assumption was that these people got the infection outside the country. Few cases were reported to be through internal transmission by the Presidential Task Force on COVID-19 (PTFC). However, the trends have changed this time around as most of the cases reported by the PTFC are internally transmitted. The current wave indicates that over 80 percent of infections are within communities with no travelling history [6]. This paper discusses some of the factors that have led to upsurge of COVID-19 cases in Malawi after the first wave eased from public health perspective.

### The upsurge of COVID-19 in Malawi - the second wave

**Low risk perception:** in the first wave of attack, when the pandemic was just reported internationally, majority of people in the country had high risk perception based on the reported virulence. Many people were able to follow the preventive measures by frequent hand washing, wearing of masks and practicing physical distancing when at public place. Uptake of preventive measures has been high in urban as compared to rural areas. Things changed when the country started reporting cases. Number of cases reported per month was few ranged from 3 to 1488 between the month of April and August 2020, mostly associated with international travelling [7]. Few internal transmissions were reported as compared to the second wave of attack. For those that got tested, positivity rate was low. In addition, case fatality rate was also very low. Risk perception was reduced in many people especially those with no history of international travelling. Majority of people started abandoning preventive measures both in urban and rural areas. The change in behavior among majority of people can best be explained using Health Belief Model [8] that explains how people may respond to a disease outbreak as time goes. We suggest that the change in behavior and reduced risk perception within population can be among the factors leading to increase in the transmission of the disease in the country. Therefore, this paper suggest that the upsurge of the pandemic can be due to low-risk perception by the majority of people as they have abandoned use of preventive measures within their communities. This has led to the upsurge of cases that have caught already struggling health system unawareness and have been overwhelmed.

**The returnees from other high-risk countries:** Malawi has its citizens in foreign neighboring countries, majority being in South Africa. The boarders of the country have been left open to allow those who may return home from other countries. These citizens, when returning back home, are screened at the border to detect if they have the virus or not. Once found with the virus, they left free to go home using public transport only advised to be on self-isolation once home. Health workers are alerted about these patients. Those screened to be negative are left free and join their families. However, some of these might have the virus that cannot be detected at an early stage. Such people pose a high risk of transmitting the virus to their immediate family members and the community. It could have been better if these returnees were first quarantined [9] for the required period of 14 days before being allowed to go home and join their families. Institutional isolation for those found positive could have been a best option as adherence for self-isolation proved to be poor because of the stigma associated with the disease [10]. The primary purpose of quarantining could be to monitor the returning residents for the duration of the incubation period, and contain any possible infections to avoid local transmission. However, this was not the case in Malawi as the returning residents were left to go back to their respective homes, hence, our argument that this has led to the upsurge of the COVID-19 in the country as it has increased internal transmission.

**Use of unchartered roots:** Malawi is a land locked country with few established boarders. The well-known borders are Mwanza, Mchinji, Mulanje-Muloza, Dedza and Karonga-Songwe borders. However, there are many non-established borders where movement of people cannot be monitored and controlled. Some people are moving into and out of the country using these non-established borders. Further, small business men are using these unchartered roots frequently to get goods and other commodities from neighboring countries like Mozambique. We assume that movement of people using uncharted roots to and from other neighboring countries have increased the transmission of the COVID-19 as they cannot be screened. It is worth noting that majority of people with the virus develops no signs and symptoms. At the same time some may develop mild signs and symptoms that can be taken rightly like any other flue because of the low knowledge about the disease etiology. With this, the internal transmission of virus increased leading to the upsurge of the pandemic in Malawi.

**Increased number of testing sites:** Malawi´s health system faces a number of challenges due to limited availability of resource in its fight against the COVID-19 transmission. After the pandemic was first reported, the country depended mainly on screening for the signs and symptoms. In addition, high fever was considered as one of the quick signs to be detected in an individual with COVID-19. There were few testing sites in the country. The only testing sites available were in the four central hospitals (Queen Elizabeth, Kamuzu, Mzuzu and Zomba), national public health laboratory in Lilongwe, college of medicine and Liverpool-Welcome Trust in Blantyre. These centres used molecular method that requires specialized training. However, during the second wave of the virus, many testing centres have been opened. The country has sourced rapid test kits for COVID-19 that can easily detect the presents of antigen in the body produced by the virus. This test is simple and takes 30 minutes for the results to come out. Almost all districts in the country are providing COVID-19 test to those presenting with signs and symptoms. Therefore, we suggest that the increased number of testing sites have increased case detection rate leading to an increase in the confirmed cases. Moreover, the new strain of the virus spread fast and causing severe signs and symptoms with high positivity rate which can also be attributed to the upsurge. This has aided the disease surveillance on positive way hence the upsurge of COVID-19 cases. Many people with signs and symptoms are being tested, detected, counted and isolated by the surveillance team in all districts in Malawi.

**Institution mass screening:** since COVID-19 second wave of attack, government ordered that schools must temporarily be closed for a period of three weeks pending further situation assessment. However, some boarding schools had already reported about the outbreak of the virus in their schools. This moved government to quickly come up with some guidelines to be followed by all boarding schools. One of the notable guidelines to be followed was screening of all students before allowing them to go back home. Further, government directed that prisons should also be screened. The presents of rapid test kits assisted health personnel to conduct screening test in prisons and boarding schools. It has already been argued that majority of COVID-19 cases are either pre-symptomatic or asymptomatic elsewhere. Only few cases are symptomatic. The mandatory screening in the institutions flagged out these asymptomatic cases in the community. We urge that this exercise assisted to detect some cases even asymptomatic ones. The country could have done it better if this was initiated during the first wave of attack especially in border posts. We therefore, attributed the upsurge of figures of COVID-19 patients to this institutional screening.

**Adherence to self-isolation and quarantine procedures:** self-isolation or quarantine is one way of limiting further spread of the disease in the community. However, monitoring and enforcement of the self-isolation or quarantine in communities is not easy in Malawi more especially in rural areas where majority of people lives. We assume that, those found positive are not following self-isolation guidelines and their contacts also do not adhere to self-quarantine protocol. This is so because of the stigma associated with the disease. As such, these high risk people, mix freely with their communities. In addition, we assume that some of the patients give false contact details to the community health workers making it difficult for contact tracing. Thus, transmission of the disease is not interrupted. We, therefore, attribute this to the high upsurge of the disease in the country. Lastly, Table 1 below summarises our recommendations and strategies that can be followed to curb the burden of COVID-19 in Malawi.

**Table 1:** recommendations and strategies to curb the burden of COVID-19 in Malawi

Recommendations	Strategies to be used
A wide community sensitization and peer to peer civic education	Use of community radios, TVs, public address systems, use of community leaders and political leaders
The country should have institutional quarantine centres	All returnees should be quarantined for 14 days before they can join their families
The country should have institutional isolation centres	All returnees who test positive should be kept at institutional isolation centres until when they test negative
Vaccination	Mass vaccination could be the lasting solution
The country to enforcing physical distance	All social activities should be restricted
The country should strengthening health system and treatment centres	To equip all isolation centres with necessary equipments like oxygen concentrators, cylinders and person protective equipments

**Conclusion:** the need to combat COVID-19 pandemic and curbing further spread of the disease in Malawi and globally cannot be over emphasized. Minimizing internal community transmission requires multi-sectoral approach so that the upsurge of the virus can be reversed. Revitalize the preventive measures in communities, public places like markets, schools, religion gathering places, funerals should be enhanced and be prioritized as a matter of urgency. Public health specialists should continue working towards finding the lasting solution that can bring the pandemic to an end. We suggest that studies focusing on preventing measures should be enhanced so that transmission should be interrupted.
